# Reference Gene Selection for Real-Time Quantitative PCR Analysis of the Mouse Uterus in the Peri-Implantation Period

**DOI:** 10.1371/journal.pone.0062462

**Published:** 2013-04-24

**Authors:** PengFei Lin, XiangLi Lan, FengLei Chen, YanZhou Yang, YaPing Jin, AiHua Wang

**Affiliations:** 1 Key Laboratory of Animal Biotechnology of the Ministry of Agriculture, Northwest A&F University, Yangling, Shaanxi, China; 2 College of Veterinary Medicine, Northwest A&F University, Yangling, Shaanxi, China; Philipps University, Germany

## Abstract

The study of uterine gene expression patterns is valuable for understanding the biological and molecular mechanisms that occur during embryo implantation. Real-time quantitative RT-PCR (qRT-PCR) is an extremely sensitive technique that allows for the precise quantification of mRNA abundance; however, selecting stable reference genes suitable for the normalization of qRT-PCR data is required to avoid the misinterpretation of experimental results and erroneous analyses. This study employs several mouse models, including an early pregnancy, a pseudopregnancy, a delayed implantation and activation, an artificial decidualization and a hormonal treatment model; ten candidate reference genes (*PPIA, RPLP0, HPRT1, GAPDH, ACTB, TBP, B2M, 18S, UBC* and *TUBA*) that are found in uterine tissues were assessed for their suitability as internal controls for relative qRT-PCR quantification. GeNorm^PLUS^, NormFinder, and BestKeeper were used to evaluate these candidate reference genes, and all of these methods identified *RPLP0* and *GAPDH* as the most stable candidates and *B2M* and *18S* as the least stable candidates. However, when the different models were analyzed separately, the reference genes exhibited some variation in their expression levels.

## Introduction

Embryo implantation is a sophisticated stage of mammalian reproduction and includes blastocyst apposition and the subsequent attachment and invasion of the uterine luminal epithelium [1]. Decidualization is critical for the establishment of fetal-maternal communication and the progression of implantation [2]. Endometrial gene expression is regulated by ovarian steroids and other paracrine molecules secreted by neighboring cells. During this process, progesterone and estrogen are the dominant hormonal modulators of endometrial development [3]. Although many specific factors are known to play a role in the implantation period [3], [4], the exact molecular mechanisms of embryo implantation remain unclear.

Real-time quantitative RT-PCR (qRT-PCR) is a reliable, accepted method for quantifying gene transcript levels. It is a fast, sensitive and accurate method for the detection of low-abundance mRNAs and slight variations in gene expression [5]. However, it requires an appropriate internal reference gene (RG) to normalize the target gene expression. In theory, such RGs must therefore exhibit constant expression levels in all cell types and experimental conditions. In fact, several studies have reported that the stability of commonly used RGs can vary between species, tissue types and experimental treatments [6]–[8]. Furthermore, previous studies have demonstrated that the conventional use of a single gene to normalize the target mRNA expression levels may produce highly inaccurate data for a significant proportion of the samples [9]. Therefore, careful selection of multiple, experimentally validated RGs is essential for accurate normalization of gene expression data [10]. The data normalization strategy for eliminating technically or experimentally induced variation is also very important. Several statistical algorithms have been developed to evaluate the expression stability of candidate RGs and to determine the minimum number of normalization factor RGs under different experimental conditions [11]–[13].

The expression stability of candidate uterine RGs under specific conditions has been measured [14]–[17], but a thorough evaluation of RG expression stability in qRT-PCR analysis of target genes expressed in the mouse uterus in conditions of early implantation has not yet been reported. Here, to identify ideal RGs, the expression levels of ten candidate RGs expressed in uterine tissues in different models of pregnancy were determined using the geNorm, NormFinder and BestKeeper software.

## Materials and Methods

### Ethics Statement

The entire experimental procedure was approved by the Committee for the Ethics on Animal Care and Experiments at Northwest A&F University. Adult male and female mice (Kunming White outbred strain) were purchased from the central animal laboratory of Xi’An JiaoTong University and kept in a temperature- (24±2°C) and light-controlled room (12 h light, 12 h darkness) with free access to food and water.

### Animals and Treatments

Mouse models of early pregnancy, pseudopregnancy, delayed implantation and activation, artificial decidualization and hormonal treatment were produced as described in previous reports [18]. The entire uterus was collected immediately following sacrifice by cervical dislocation and was then stored at −80°C until further analysis.

### Tissue RNA Extraction and cDNA Synthesis

Total RNA was extracted from all tissues using Trizol (Invitrogen, Inc., CA) according to the manufacturer’s instructions and was then treated with DNase (TaKaRa Bio, Inc., Dalian, China) to remove genomic DNA contamination prior to RT. The extracted RNA was dissolved in diethypyrocarbonate (DEPC)-treated water, and the RNA concentration and purity were estimated by reading the absorbance at 260 and 280 nm on a spectrophotometer (Eppendorf, Inc., Hamburg, Germany). The absorption ratios (260/280 nm) for all preparations were between 1.8 and 2.0. Aliquots of the RNA samples were subjected to electrophoresis using a 2% agarose gel to verify their integrity. Samples with a 28S/18S ribosomal RNA ratio between 1.5 and 2.0 without smears on the agarose gel were used for the following experiment. cDNA was synthesized using a PrimeScript™ RT reagent Kit (TaKaRa Bio, Inc., Dalian, China) according to the manufacturer’s instructions. The final volume of each experimental reaction was 20 µl, which included 400 ng of total RNA.

### Candidate RGs, Primer Design and Amplicon Specificity

Ten candidate RGs were evaluated in this study: *PPIA, RPLP0, HPRT1, GAPDH, ACTB, TBP, B2M, 18S, UBC* and *TUBA* (Table 1). Primers for these genes were selected according to PrimerBank (http://pga.mgh.harvard.edu/primerbank), except for the *GAPDH, ACTB* and *TUBA* primers, which were designed by the Primer 5.0 software. All primers were confirmed using the NCBI Blast tool to compare them against all available mRNA sequences to ensure their specificity. The sequences for each set of primers are listed in Table 2. The efficiency (*E*) of each primer pair was determined across a range of standard dilutions (using a minimum 5 log range) and calculated according to the formula *E* = 10^1/slope^. Only Ct values of less than 40 were used to calculate the correlation coefficients (r^2^ values). All primer pairs used in this study had an efficiency greater than 95%.

**Table 1:** Summary of candidate reference genes used in this study.

Gene symbol	Gene name	Gene function
PPIA	Peptidylprolyl isomerase A	Catalyzes the cis-trans isomerization of proline imidic peptide bonds in oligopeptides and accelerates the folding of proteins.
RPLP0	Ribosomal protein, large, P0	Structural constituent of ribosome.
HPRT1	Hypoxanthine-guanine phosphoribosyltransferase 1	Purine synthesis in salvage pathway.
GAPDH	Glyceraldehydes-3-phosphate dehydrogenase	Glycolytic enzyme.
ACTB	Actin, beta	Formation of major component of the cytoskeleton.
TBP	TATA box binding protein	Composed of transcription factor IID with TBP-associated factors.
B2M	Beta-2-microglobulin	Major histocompatibility complex.
18S	Ribosomal protein 18s	Central component of the ribosome. Provides a mechanism for decoding mRNA into amino acids.
UBC	Ubiquitin C	Possible involvement in protein catabolism.
TUBA	Tubulin α-1	Microtubules of the eukaryotic cytoskeleton.

**Table 2:** Primer information for qRT-PCR amplification.

Gene symbol	Accession number	Primer sequence (5′ to 3′)	Amplicon length (bp)	PCREfficiency (%)
PPIA	NM_008907	CAAATGCTGGACCAAACACAAACG GTTCATGCCTTCTTTCACCTTCCC	110	95.6
RPLP0	NM_007475	GGACCCGAGAAGACCTCCTT GCACATCACTCAGAATTTCAATGG	85	103.5
HPRT1	NM_013556	TCAGTCAACGGGGGACATAAA GGGGCTGTACTGCTTAACCAG	142	105.6
GAPDH	NM_008084	TCACTGCCACCCAGAAGA GACGGACACATTGGGGGTAG	186	101.7
ACTB	NM_007393	GCAAGCAGGAGTACGATGAG CCATGCCAATGTTGTCTCTT	148	104.9
TBP	NM_013684	GGCCTCTCAGAAGCATCACTA GCCAAGCCCTGAGCATAA	107	101.8
B2M	NM_009735	ATTCACCCCCACTGAGACTG TGCTATTTCTTTCTGCGTGC	193	96.7
18S	NR_003278	CTCAACACGGGAAACCTCAC CGCTCCACCAACTAAGAACG	110	105
UBC	XM_001471699	AGCCCAGTGTTACCACCAAG ACCCAAGAACAAGCACAAGG	97	103.3
TUBA	NM_011653	CCAGGGCTTCTTGGTTTTCC CTACCATGAAGGCACAATC	218	102.7

### qRT-PCR

qRT-PCR was performed using three biological replicates and technical triplicates/duplicates of each cDNA sample using the SYBR® Premix Ex Taq™ II Kit (TaKaRa Bio, Inc., Dalian, China) with the Multicolor Real-Time PCR detection system (iQ5, Bio-Rad Laboratories, Inc., Hercules, USA), according to the manufacturer’s protocol. Each PCR reaction (with a total volume of 20 µl) consisted of 2 µl reverse transcription product, 0.8 µl each of the 10 µM forward and reverse primers, 10 µl SYBR® Premix Ex Taq™ II, and 6.4 µl RNase-free water. Cycling conditions consisted of a denaturation step at 95°C for 30 sec, followed by 45 PCR cycles of 95°C for 5 sec and 60°C for 20 sec. A melting curve analysis was performed at the end of each PCR program to prevent nonspecific product formation. The resulting PCR products (for each gene and in each sample) were run on an agarose gel to ensure the specificity of the amplification.

### Statistical Analyses

All the experiments were independently repeated at least three times. For each group, the uterine tissues were collected from the three individual animals at each tissue collection time point. The data were accessed from the iQ5 Optical System Software v2.0 software with auto thresholds and baselines in all experiments. The cycle threshold (Ct) was read from the interpolation of the threshold level and the amplification curve. The expression stability of candidate RGs across the various experimental treatments was analyzed using three different mathematical algorithms: geNorm^PLUS^ (http://medgen.ugent.be/~jvdesomp/genorm/) [11], NormFinder (http://www.mdl.dk/publicationsnormfinder.htm) [12], and BestKeeper (http://gene-quantification.com/bestkeeper.html) [13]. All three software packages were used according to the manufacturer’s instructions.

## Results

### Primer Specificity and Efficiency Analyses

Due to the uniformity of the initial primer selection criteria, it was possible to use similar reaction conditions for all primers in the qRT-PCR assays. The amplification specificity of several transcripts was confirmed using melting curve analysis and agarose gel electrophoresis, which showed a single peak for each unique amplification product of the expected size for each gene. The primer efficiencies were in the recommended range (90–105%), except for the *HPRT1* primers, which had a slightly higher amplification efficiency (105.6%, Table 2). Altogether, these results confirmed that the selected primers accurately amplify candidate reference genes.

### Gene Expression Profile Analyses

**Figure 1:**
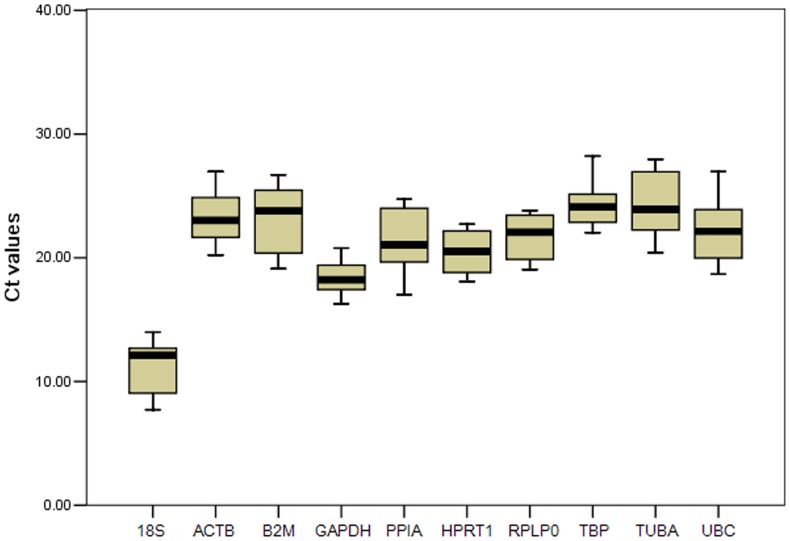
Range of Ct values for the candidate RGs obtained from all cDNA samples. Each box corresponds to 18S, ACTB, B2M, GAPDH, PPIA, HPRT1, RPLP0, TBP, TUBA and UBC and indicates the 25% and 75% percentiles. Whiskers represent the maximum and minimum values. The median is depicted as a line across the box.

The calculated mean Ct values for the 10 RGs in all the cDNA samples ranged from 7.72 to 28.22 (Fig. 1). Of the tested candidates, the transcript abundance was highest for *18S* and lowest for *TUBA*. For the *UBC* gene, a larger variation was found (18.67≤Ct≤26.96). Based on the interquartile range (between the 25–75% percentiles) for the Ct values, a lower Ct dispersion was observed for *GAPDH, TBP, RPLP0, HPRT1, ACTB*, and *UBC*, followed by *18S, PPIA, TUBA*, and *B2M*. These results suggest that *GAPDH* is the most stable gene in terms of its mRNA expression levels and that *B2M* is not suitable for normalizing gene expression during combined sample analysis.

### GeNorm Analysis

**Figure 2:**
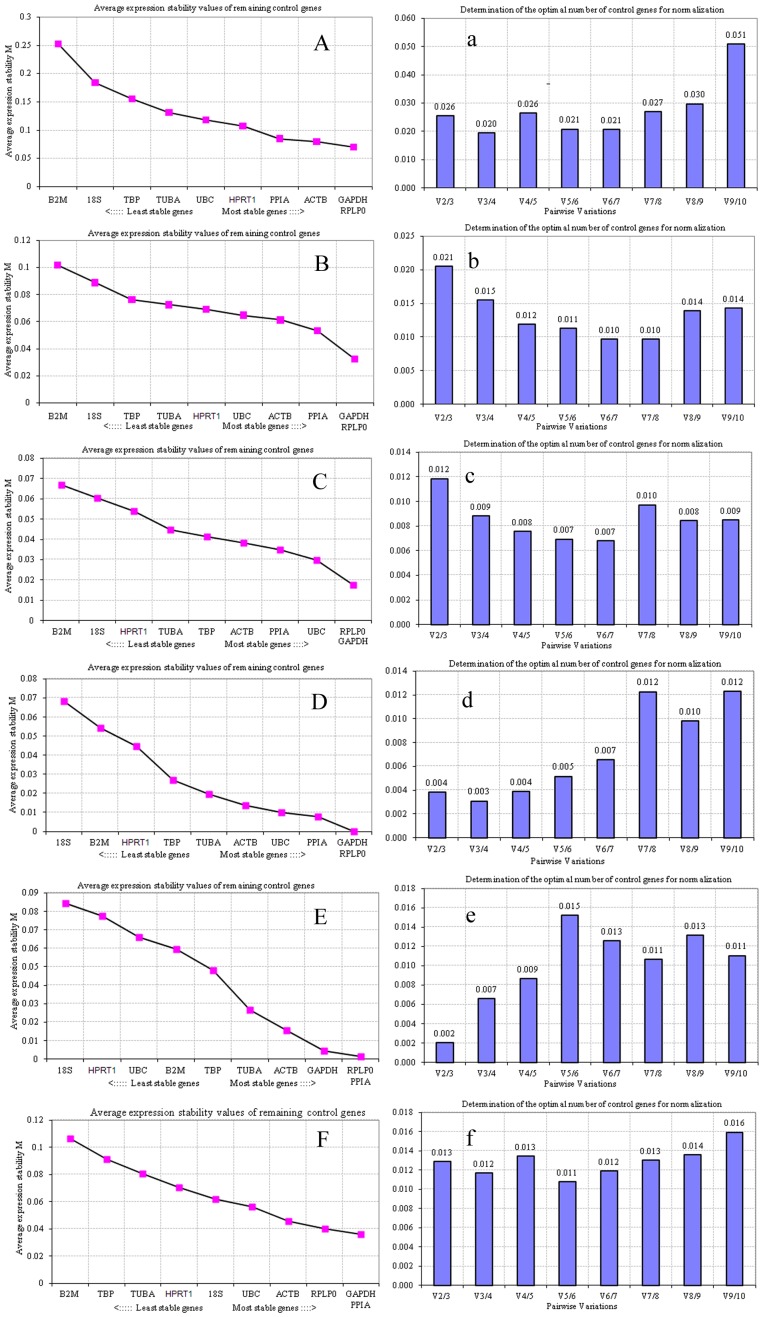
GeNorm analysis of the expression stability of the 10 RGs. (A–F) Average expression stability values (M) and ranking of the candidate RGs as calculated by geNorm software. Lower M values indicate more stable expression. (a–f) Determination of the optimal number of RGs for normalization by pairwise variation (V) as assessed by geNorm. According to the algorithm and software instructions, a cut-off V value of 0.15 was used.

When conducting a geNorm analysis, the gene with the lowest M value has the most stable expression, while the gene with the highest M value has the least stable expression [11]. Considering the data obtained from all samples of the early pregnant mouse uterus, *GAPDH* and *RPLP0* were found to be the two most stable genes with the lowest M values. Similarly, *B2M* was found to be the least stable gene with the highest M value (Fig. 2A and B). During pseudopregnancy, the *GAPDH* and *RPLP0* genes showed the greatest expression stability, while *B2M* showed the least expression stability (Fig. 2C). As shown in Figure 2D, *RPLP0* and *GAPDH* were the most stably expressed genes under conditions of delayed and activated implantation, while *18S* was the least stably expressed. In the artificial decidualization model, *RPLP0* and *PPIA* were expressed more stably than the other eight RGs, while *18S* was expressed less stably than all other RGs (Fig. 2E). Under conditions of hormonal treatment, *PPIA* and *GAPDH* were the most stably expressed genes, while *B2M* was the least stably expressed gene (Fig. 2F). Overall, all tested RGs had relatively high stability with low M values of less than 0.30, which is below the default limit of M<0.5 [16]. Evaluation of all the expression data revealed that *B2M* and *18S* were the least stably expressed reference genes and that *RPLP0, GAPDH* and *PPIA* were the most stably expressed reference genes. Thus, *RPLP0, GAPDH* and *PPIA* may be suitable for gene expression analysis in the five model systems used in this study.

In addition, geNorm was used to calculate the number of optimal RGs for deriving a normalization factor (NF). Vandesompele *et al.* [11] proposed 0.15 as the cut-off value for V, below which the inclusion of additional RGs is not required. Thus, if V_n/n+1_<0.15, it is not necessary to use ≥n+1 RGs as internal controls. Accordingly, the pairwise variation of V2/3 was lower than 0.15 in all of the conditions (Fig. 2a–f), demonstrating that the combined use of the two most stable RGs would be sufficient to normalize gene expression in all uterine models used in this study.

### NormFinder Analysis

The results of the NormFinder analysis are reported in Table 3. As with the geNorm method, the gene with the lowest M value has the most stable expression, and the gene with the highest M value has the least stable expression. When the results from all uterine samples were combined during early pregnancy, the two most stable (*GAPDH* and *RPLP0*) and the two least stable (*B2M* and *18S*) candidate genes were the same as those identified by geNorm; however, a different ranking of the remaining candidate RGs was obtained. Using the artificial decidualization model, *GAPDH* and *RPLP0* were found to be the most stably expressed genes, and *18S* was found to be the least stably expressed gene. *RPLP0* and *PPIA* were the most stably expressed genes, and *B2M* was the least stably expressed gene in the pseudopregnancy, delayed implantation and hormonal treatment models.

**Table 3:** Ranking of candidate reference genes in order of their expression stability as calculated by NormFinder.

Gene	Experimental models					
	All	Early Pregnancy	Pseudopregnancy	Delayed and activatedimplantation	Artificial decidualization	Hormonal treatments
PPIA	0.054	0.041	**0.028**	**0.014**	0.022	**0.029**
RPLP0	**0.053**	**0.028**	**0.028**	**0.024**	**0.011**	**0.015**
ACTB	0.061	0.047	0.030	0.026	0.022	0.046
GAPDH	**0.043**	**0.031**	0.032	0.027	**0.010**	0.038
UBC	0.099	0.055	0.034	0.033	0.052	0.030
TBP	0.197	0.034	0.037	0.034	0.044	0.071
HPRT1	0.098	0.034	0.042	0.037	0.073	0.050
TUBA	0.110	0.050	0.044	0.045	0.040	0.053
18S	0.162	0.064	0.047	0.059	***0.082***	0.064
B2M	***0.330***	***0.089***	***0.059***	***0.074***	0.068	***0.094***

Note: Data were presented as expression stability value (M value) as calculated by NormFinder. Lower average expression stability (M value) indicates more stable expression. The values in bold refer to the most stable genes, and the values in bold italic refer to the least stable genes.

### BestKeeper Analysis

BestKeeper was also used to calculate the coefficient of variance (CV) and the standard deviation (SD) of the Ct values using the whole data set, and all Ct values were analyzed as a total data set [13]. The most stable RGs were identified based on having the lowest coefficient of variance and standard deviation (CV±SD). In this study, *GAPDH* and *RPLP0* were found to have remarkably stable expression in all the samples, while *B2M* was found to have the least stable expression (Table 4). These results were consistent with those obtained from geNorm and NormFinder (Fig. 2A and Table 3). BestKeeper analyses indicated that *RPLP0* and *PPIA* were the most stably expressed and that *B2M* was the least stably expressed in the early pregnant and pseudopregnant uteruses. In the delayed and activated implantation scenarios, the most stable RGs were *RPLP0* and *ACTB*, and *18S* had the highest CV±SD of all of the selected genes. *PPIA* and *RPLP0* were the most stably expressed genes, and *18S* had the lowest CV±SD under artificial decidualization. In the hormone-treated models, the most stable genes were found to be *GAPDH* and *RPLP0*, and the least stable gene was found to be *B2M*.

**Table 4:** Ranking of candidate reference genes in order of their expression stability as calculated by BestKeeper.

Gene	Experimental models					
	All	Early Pregnancy	Pseudopregnancy	Delayed and activated implantation	Artificialdecidualization	Hormonal treatments
RPLP0	**1.22±0.36**	**1.08±0.15**	**0.74±0.11**	**0.54±0.16**	**1.05±0.33**	**1.15±0.63**
UBC	1.76±0.45	2.41±0.90	1.19±0.24	1.77±0.41	1.17±0.30	1.45±0.51
TUBA	1.86±0.72	1.43±0.80	2.16±0.87	1.34±0.62	2.15±0.83	2.52±0.72
PPIA	1.48±0.56	**1.09±0.96**	**0.86±0.26**	1.44±0.32	**0.47±0.12**	1.21±0.43
ACTB	1.48±0.91	1.98±0.48	1.73±0.61	**1.20±0.85**	1.62±0.19	1.30±0.44
GAPDH	**1.14±0.35**	1.17±0.27	0.95±0.81	1.25±0.22	1.06±0.82	**1.01±0.95**
HPRT1	1.81±0.64	1.19±0.76	2.23±1.07	2.13±0.69	2.09±0.53	2.82±0.58
TBP	2.88±0.94	2.24±0.46	1.76±0.79	2.65±0.53	2.21±0.79	2.68±0.89
B2M	***3.17±0.86***	***2.84±0.42***	***2.24±0.63***	3.85±0.93	2.62±0.76	***3.09±1.09***
18S	2.78±0.99	2.46±0.95	1.85±0.26	***3.94±0.60***	***2.80±1.12***	2.67±0.59

Note: Data were presented as coefficient of variance (CV) ± standard deviation (SD). Reference genes are identified as the most stable genes (those with the lowest CV ± SD value). The values in bold refer to the most stable genes, and the values in bold italic refer to the least stable genes.

## Discussion

Blastocyst implantation and the successful establishment of pregnancy depend on delicate interactions between the embryo and the surrounding maternal environment. Pseudopregnancy, delayed implantation, artificial decidualization and hormonal treatment in mice are important models used for the investigation of the physiological and molecular events necessary for the implantation process [19]. Gene expression profiles of these models at the mRNA and protein level are extremely valuable for further elucidation of the molecular mechanisms involved in implantation. qRT-PCR is a convenient and accurate method to assess target gene mRNA expression during embryo implantation. However, the selection of RGs used can significantly influence the result obtained. Although many stable RGs from preimplantation embryos have been screened [6], no publication to date has validated these uterine RGs for the implantation process. In this study, we analyzed the expression of 10 candidate RGs using three popular software packages: geNorm, NormFinder and BestKeeper. These packages have been widely used in RG selection and have been previously utilized in studies associating gene function with the reproductive process. GeNorm is the most commonly used method to determine the optimal number of stable RGs required for data normalization, while NormFinder and BestKeeper are often used to assess the quality of the geNorm rankings [20]. The expression stability M-value of all candidate RGs was less than 1.5 according to the geNorm analysis, indicating that these candidate genes were suitable for use as endogenous control genes due to their stable expression.

In combination, the analysis results of these three software packages indicate that *RPLP0*, *GAPDH* and *PPIA* are the most stable RGs and can therefore serve as control RGs for future studies of the mouse uterus during implantation (Table 5). Although *ACTB* was found to be one of the best candidate genes in the delayed and activated implantation model as assessed by BestKeeper analysis, this result was not consistent with that obtained using NormFinder and geNorm. In our data analysis, determination of the pairwise variation of two sequential normalization factors (Vn/n+1) with the geNorm software indicated that a minimum of two RGs should be included in the normalization process for all experimental models under study. Here, geNorm and NormFinder identified *RPLP0* and *GAPDH* as the most stable RGs in the early pregnant uterus, while BestKeeper indicated that *PPIA* was actually a more stable RG than *GAPDH*. The top two RGs for the artificial decidualization model predicted by geNorm were similar to those determined by BestKeeper but differed from those determined by NormFinder. Furthermore, the three software packages predicted different optimum RG pairs for the delayed implantation and hormonal treatment models. Such significant differences in the optimal RG predictions of the three software packages are likely due to the different calculation algorithms each package uses. However, because all three software packages identified *RPLP0* and *GAPDH* as the most stable RGs for all the samples, it seems likely that the combination of *RPLP0* and *GAPDH* would be optimal for further analysis.

**Table 5:** Comparison of reference gene expression as determined by three distinct algorithms.

Experimental models	The most stable genes			The least stable genes		
	GeNorm	NormFinder	BestKeeper	GeNorm	NormFinder	BestKeeper
All	RPLP0 & GAPDH	GAPDH & RPLP0	GAPDH & RPLP0	B2M	B2M	B2M
Early Pregnancy	RPLP0 & GAPDH	RPLP0 & GAPDH	RPLP0 & PPIA	B2M	B2M	B2M
Pseudopregnancy	GAPDH & RPLP0	RPLP0 & PPIA	RPLP0 & PPIA	B2M	B2M	B2M
Delayed and activatedimplantation	RPLP0 & GAPDH	PPIA & RPLP0	RPLP0 & ACTB	18S	B2M	18S
Artificial decidualization	PPIA & RPLP0	GAPDH & RPLP0	PPIA & RPLP0	18S	18S	18S
Hormonal treatments	PPIA & GAPDH	RPLP0 & PPIA	GAPDH & RPLP0	B2M	B2M	B2M

Tissue-specific RG expression profiles have been reported for 15 different mouse tissues. These were generated using the serial analysis of gene expression (SAGE) strategy, which identified significant differences in the expression levels of RGs such as *PPIA* and *GAPDH* [8]. Previous studies have shown that the day of pregnancy may affect the stability of RG expression in both fetal and adult tissues [21], [22]. We demonstrate here that pregnancy may also influence RG expression in maternal uterine tissues. *GAPDH* is currently one of the most commonly used RGs for normalizing gene expression data in qRT-PCR assays. *GAPDH* has been traditionally used in studies of the mouse uterus during embryo implantation [18], [23]; however, experimental validation had not been performed. Kayis *et al.* [16] reported that *GAPDH* was the most stable RG and *18S* the least stable RG in the early pregnant equine endometrium, which is consistent with the results of our study. However, *GAPDH* was found to have less stable expression in the bovine endometrium during early pregnancy [17]. Therefore, the expression level of RGs varies between different species. The l RG of *RPLP0* had little or no variation across the different experimental models used in this study, as assessed by the three software packages. *RPLP0* has also been found to have highly stable expression in all ovarian-related research to date [24]. It is therefore likely that of the RGs assessed in this study, *RPLP0* is the best RG for gene expression evaluation in mouse uteruses, and *GAPDH* is the second best RG for gene expression evaluation.

*18S* is commonly used as a control in gene expression studies because it is abundantly expressed in all cell types. Previously, *18S* was reported to be stably expressed in the hypothalamus between the pre- and postnatal periods in mice [25]. Unexpectedly, while *18S* was the most abundant of the included candidate RGs from the uterine samples, all three software packages ranked *18S* in the bottom position. Similar analyses from all three software packages ranked *B2M* as the least stable gene. These results suggest that *B2M* and *18S* may not be optimal genes to use as RGs in the mouse uterus during embryo implantation.

In summary, this study provides the first reported assessment of endogenous control genes for use in expression studies in the peri-implantation mouse uterus. The different experimental models and software packages used to identify expression stability did not rank the candidate RGs in the same order. In general, the results of this study indicate that *RPLP0* is a better choice than *GAPDH* when using a single RG to assess target gene expression. It is recommended that a normalization factor derived from *RPLP0* and *GAPDH* be used when qRT-PCR is performed in various experimental models of the mouse uterus.
